# Supplementary material for: The Effect of Developmental Pleiotropy on the Evolution of Insect Immune Genes
Source: Genome Biol Evol. 2023 Mar 13;15(3):evad044. doi: 10.1093/gbe/evad044 (PMC10063218; doi:10.1093/gbe/evad044)
Supplement: evad044_Supplementary_Data [file evad044_supplementary_data.zip › 17Jan2023_Supplemental_File_2_clean.docx]

**Supplemental File 2**

The effect of developmental pleiotropy on the evolution of insect immune genes

Alissa M. Williams^, Thi Minh Ngo^, Veronica E. Figueroa, and Ann T. Tate*

Department of Biological Sciences and Evolutionary Studies Initiative, Vanderbilt University, Nashville, TN, USA

*Author for Correspondence: Ann Tate, Department of Biological Sciences and Evolutionary Studies Initiative, Vanderbilt University, Nashville, TN, USA. Email: [a.tate@vanderbilt.edu](mailto:a.tate@vanderbilt.edu)

^ These authors contributed equally to this work.

**Supplemental Methods**

**Developmental and Immune Gene curation**

Genes associated with particular GO terms are obtained from the FlyBase GO QuickSearch tool. For each of the GO terms considered, the list of gene IDs in FBgn format was downloaded, and grouped together according to Supplemental Figure 1.

**Immune Gene Class assignment**

First, we curated a list of all immune genes that lack a gene class assignment from previous studies. This list was then used to queried in the FlyBase “Query by symbol” tool, and gene description are obtained from the field “Gene Snapshot” and “GO_Molecular”, which was used to assign each gene to categories such as Recognition, Signaling and Effector, if available. Note that genes belonging to "Other" are those that do not seem to belong to a signaling pathway, but still play important roles in immune response, such as structural binding complexes, autophagy regulators or cell migration complexes. Those that are in "Signaling" are either modulators of well-known pathways or direct participants of the pathway.

**FlyBase queries**

The following files from the FlyBase release (http://ftp.flybase.net/releases/FB2020_02/) were used for analysis in this study:

dmel_unique_protein_isoforms_fb_2020_02.tsv.gz gene_rpkm_report_fb_2020_02.tsv.gz gene_snapshots_fb_2020_02.tsv.gz
gene_association.fb.gz dmel_orthologs_in_drosophila_species_fb_2020_02.tsv.gz

**Pathway analysis:**

To query the pathways associate with each gene group, we used PANTHER (http://www.pantherdb.org/). The gene IDs associated with each group was entered and the list of associated pathways was downloaded.

Toll and IMD pathway:

https://www.genome.jp/kegg-bin/show_pathway?dme04624

**Downsampling:**

**Tau values:**

To account for the potential effect of sample size, we downsampled the τ distributions in Figure 2A by bootstrapping each gene class to the same number of genes (we used the smallest number of genes, which was 162 in the pleiotropic class). We completed this process 100 times for each gene class, calculated the medians of these distributions, and then plotted the medians (Supplemental Figure 5). These results were consistent with those described above; the median values of these bootstrapped median distributions are 0.732, 0.669, and 0.692 for non-pleiotropic immune genes, pleiotropic genes, and non-pleiotropic developmental genes, respectively. These values are all statistically different from one another (Kruskal-Wallis w/Dunn test, p.adj < 0.001).

***d_N_/d_S_* values:**

As with the τ values, we downsampled the *d_N_/d_S_* distributions for the 12 species dataset to have the same number of genes each (we downsampled to the number of genes in the smallest class, which was the pleiotropic class). We downsampled using bootstrapping 100 times for each gene class, calculated the median of each of the bootstrap replicates, and plotted the distribution of medians (Supplemental Figure 6). The median of this distribution was 0.085 for non-pleiotropic immune genes, 0.063 for pleiotropic genes, and 0.064 for non-pleiotropic developmental genes; these three categories had significantly different distributions based on a Kruskal-Wallis test (Supplemental Figure 6, χ² = 199.53, p < 2.2e-16). The comparison between pleiotropic genes and developmental non-pleiotropic genes does not show a statistically significant difference (p = 0.31), but non-pleiotropic immune genes have a statistically different distribution relative to both non-pleiotropic developmental genes (p < 2.2e-16) and pleiotropic genes (p < 2.2e-16).

We also completed downsampling via bootstrapping on the 6 species dataset to account for sample size discrepancies; we downsampled each gene class to the number of genes in the pleiotropic class 100 times and plotted the distribution of median values across bootstrap replicates (Supplemental Figure 7B). The medians of these distributions were 0.078 for non-pleiotropic immune genes, 0.061 for pleiotropic genes, and 0.058 for non-pleiotropic developmental genes; these three categories had significantly different distributions based on a Kruskal-Wallis test (Supplemental Figure 7B, χ² = 208.28, p < 2.2e-16). All pairwise comparisons between distributions were statistically significant in this case (p < 2.2e-16).

***α, ω_a_,* and *ω_na_* values:**

We performed bootstrap replication in a second way to account for potential effects of sample size discrepancies. Instead of summing the n site frequency spectra, where n = the number of genes in that category in that population, we summed the same number of site frequency spectra for all three classes within each population (set to the number of pleiotropic genes, since that was the smallest number). With this second form of bootstrapping, median values of *α* for the non-pleiotropic immune, pleiotropic, and non-pleiotropic developmental gene classes were 0.635, 0.778, and 0.722 (Supplemental Figure 8A). Median values of *ω_a_* were 0.161, 0.182, and 0.156 (Supplemental Figure 8B). Median values of *ω_na_* were 0.092, 0.052, and 0.060 (Supplemental Figure 8C). Distributions of *α*, *ω_a_,* and *ω_na_* values were significantly different across all three gene categories in both populations based on Kruskal-Wallis tests (all tests had p ≤ 4.765e-09). All comparisons between sets of two gene classes for *α*, *ω_a_,* and *ω_na_* values in both populations were significantly different using post-hoc Dunn tests with a Bonferroni correction with the exception of the comparison between the *ω_a_* values for the non-pleiotropic immune and non-pleiotropic developmental classes.

**
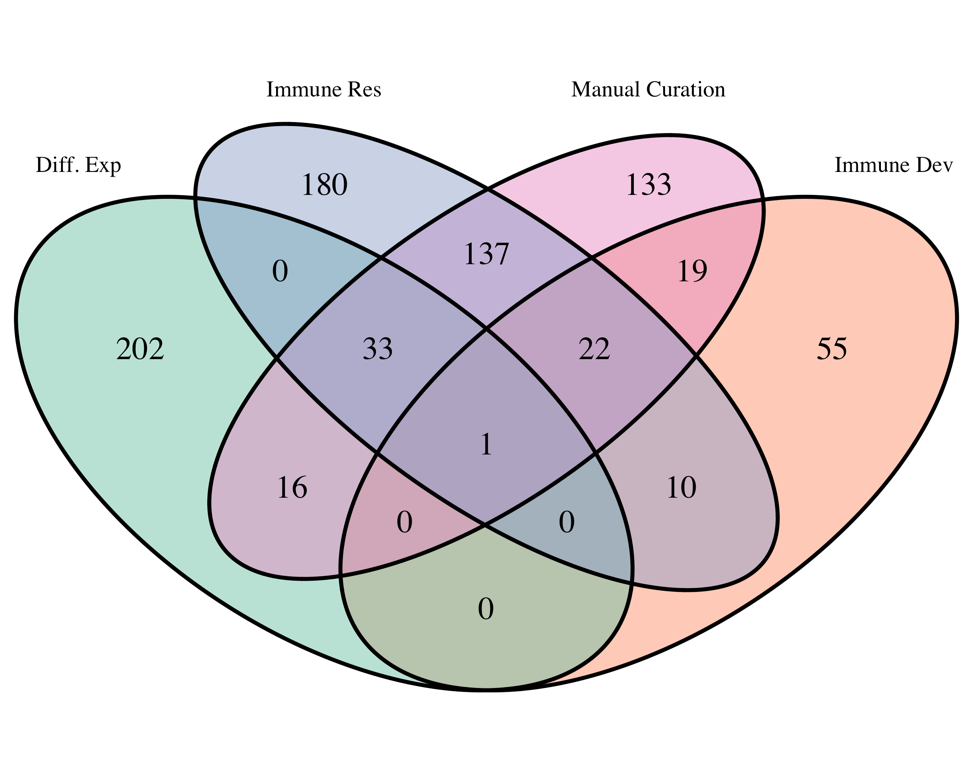
**

**Supplemental Figure 1**: Venn Diagram representing the overlap between sources used to curate the immune gene list. Four distinct lists are combined to obtain a list of 808 immune genes:

1. “Diff Exp” group refers to the core set of differentially expressed gene identified in Troha et al 2018.
2. “Immune Res” group refers to genes annotated under the following GO Terms that represent the core actions of an immune response:
   1. GO:0002252 Immune Effector Process
   2. GO:0002253 Activation Of Immune Response
   3. GO:0006955 Immune Response
   4. GO:0019882 Antigen Processing
   5. GO:0045321 Leukocyte Activation
   6. GO:0035172 Hemocyte Proliferation

Each GO term is queried in FlyBase and all six lists are concatenated, and duplicates are removed.

1. “Manual Curation” group refers to a manually curated immune gene in Early et al 2018, which also cites previous experimental studies on *Dmel* immunity.
2. “Immune Dev” group refers to genes annotated under the following GO Terms that represent genes having a role in the *development* of the immune system instead of direct participation in the immune response:
   1. GO:0002520 Immune System Development
   2. GO:0042386 Hemocyte Differentiation

Each GO term is queried in FlyBase and all six lists are concatenated, and duplicates are removed. The detailed description of each GO Term can be viewed in FlyBase.

Note that throughout the study, “Immune Response” genes refer to those in all the above groups except “Immune Development”, unless a gene is annotated under both the “Immune Res” and “Immune Dev” GO list. A main assumption and caveat to keep in mind is that differential expression may or may not represent a real participation in immunity but rather a physiological effect from infection. Since we are using a list of DE genes across infection with 10 bacteria species, we believe this list represents the core regulation of the physiological response to infection and have reduced noise compare to usage of only one dataset on infection with only one pathogen species.

**
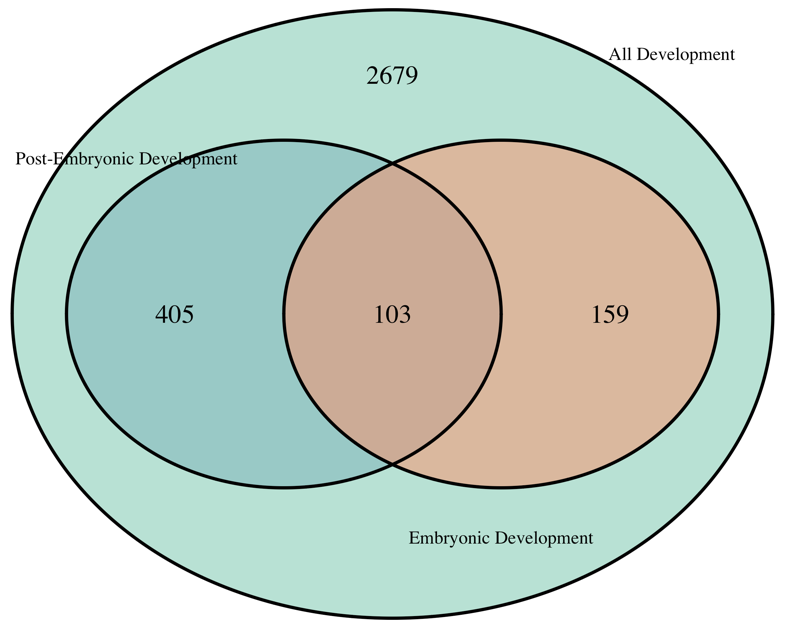
**

**Supplemental Figure 2:** Venn Diagram representing the overlap between sources used to curate the developmental gene list. Similar to the method that we used to curate the immune gene list described above, three separate GO Terms are queried through FlyBase and the list of developmental gene is annotated in accordance to their GO Term annotation:

1. GO:0032502 Developmental Process
2. GO:0048598 Embryonic Morphogenesis
3. GO:0009886 Post-embryonic Development

Throughout the study, “developmental gene” refers to all 3346 genes, unless otherwise noted. The detailed description of each GO Term can be viewed in FlyBase.


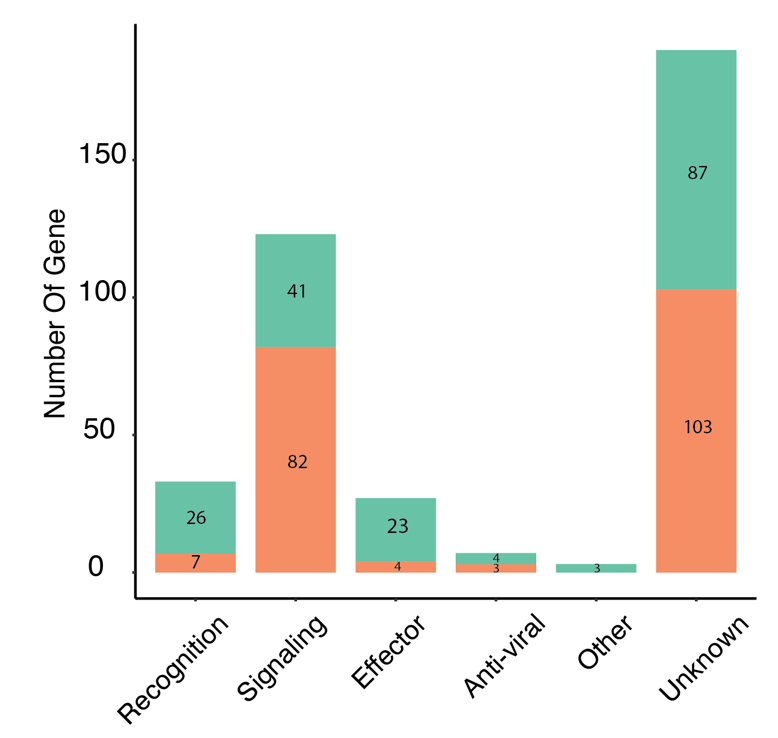


**Supplemental Figure 3:** Number of pleiotropic (orange) and non-pleiotropic (green) genes in each immune gene list.


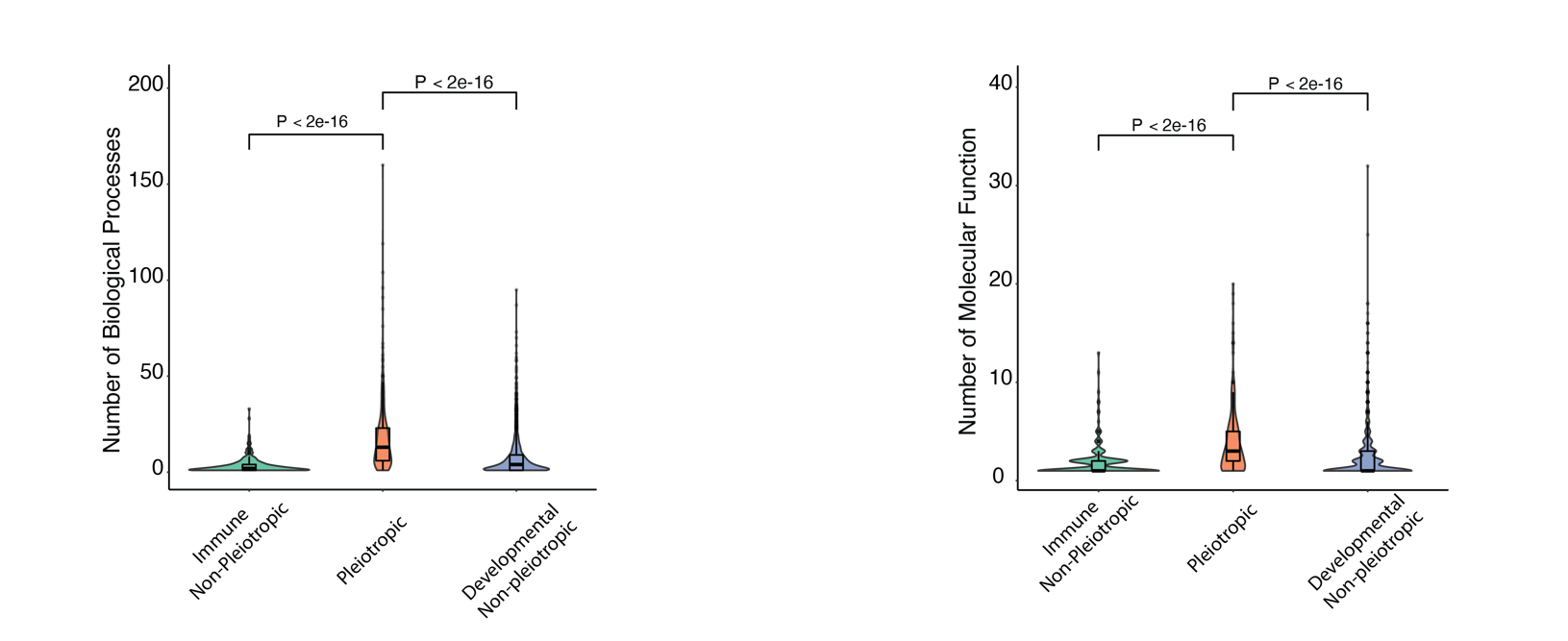


**Supplemental Figure 4:** Number or Biological Processes and Molecular Function GO terms associated with genes belonging to each pleiotropy group.

**Supplemental Figure 5:** Distributions of median Tau values across groups. Medians were calculated 100 times for each gene class using bootstrapping. The number of genes included in each bootstrap replicate was the smallest number of genes (n = 162, the number of genes in the pleiotropic class). Distributions were compared using a Kruskal-Wallis test followed by post-hoc Dunn tests in R.

Supplemental Figure 6: Downsampling of Figure 3A. All gene categories were downsampled 100 times (using bootstrapping) to get to equal sample sizes (n = 227 for all). Distributions of medians were compared using a Kruskal-Wallis test followed by post-hoc Dunn tests in R.

Supplemental Figure 7: dN/dS values for the 6 species phylogeny (melanogaster group). A) dN/dS for all genes in each class (n = 360, 257, and 2236, respectively). B) All gene categories were downsampled (using bootstrapping) to get to equal sample sizes (n = 257 for all) 100 times. Distributions of medians were compared using a Kruskal-Wallis test followed by post-hoc Dunn tests in R.

**Supplemental Figure 8:** Downsampled version of Figure 4**.** Distributions in the *Drosophila melanogaster* Raleigh (RAL) population of A) *α* values, B) *ω_a_* values and C) *ω_na_* values. *α_,_ ω_a_*, and *ω_na_* values were calculated using MultiDFE on 100 bootstrap replicates of summed site frequency spectra (SFS) for each gene category (in this case, the number of genes summed into the SFS was the same across gene classes: 207). Distributions were compared using a Kruskal-Wallis test followed by post-hoc Dunn tests in R.
