# Supplementary material for: The Effect of Developmental Pleiotropy on the Evolution of Insect Immune Genes
Source: Genome Biol Evol. 2023 Mar 13;15(3):evad044. doi: 10.1093/gbe/evad044 (PMC10063218; doi:10.1093/gbe/evad044)
Supplement: evad044_Supplementary_Data [file evad044_supplementary_data.zip › List of Supplemental Tables and Figures.docx]

**List of Supplemental Tables and Figures:**

**In Supplemental File 1:**

**Supplemental Table 1:** Categorization of each gene included in analysis according to different definitions of developmental pleiotropy as described in Table 1

**Supplemental Table 2:** Genes manually annoted for function derived from FlyBase (see Methods; Comparison of pleiotropic and non-pleiotropic immune gene characteristics)

**Supplemental Table 3:** Assignment of pleiotropic and non-pleitropic genes to PANTHER pathways

**Supplemental Table 4:** Corresponding number of paralogs among Drosophila species for each Dmel gene

**Supplemental Table 5:** The number and percentage of specific pleiotropic and non-pleiotropic genes that showed maximum expression in each stage

**Supplemental Table 6:** Full dataset used in statistical analysis of tau results

**In Supplemental File 2:**

**Supplemental Methods:** Includes methods sections for Developmental and Immune Gene curation, Immune Gene Class assignment, FlyBase queries, Pathway analysis, and Downsampling (for tau values, *d_N_/d_S_* values, and *α, ω_a_,* and *ω_na_* values).

**Supplemental Figure 1:** Venn Diagram representing the overlap between sources used to curate the immune gene list.

**Supplemental Figure 2:** Venn Diagram representing the overlap between sources used to curate the developmental gene list.

**Supplemental Figure 3:** Number of pleiotropic and non-pleiotropic genes in each immune gene list.

**Supplemental Figure 4:** Number or Biological Processes and Molecular Function GO terms associated with genes belonging to each pleiotropy group.

**Supplemental Figure 5:** Distributions of median Tau values across gene categories after downsampling to get equal sample sizes.

**Supplemental Figure 6:** Downsampled version of Figure 3A. Distributions of median *d_N_/d_S_* values across gene categories.

**Supplemental Figure 7:** *d_N_/d_S_* values for the 6 species phylogeny (melanogaster group). A) including all genes and B) after downsampling to get equal sample sizes across gene categories.

**Supplemental Figure 8:** Downsampled version of Figure 4. Distributions in the *Drosophila melanogaster* Raleigh (RAL) population of A) *α* values, B) *ω_a_* values and C) *ω_na_* values after downsampling to get equal sample sizes across gene categories in each population.
